# Tetracycline fluorescence in experimental tumours.

**DOI:** 10.1038/bjc.1965.66

**Published:** 1965-09

**Authors:** E. Tapp, R. Carroll, K. Kovács

## Abstract

**Images:**


### TETRACYCLINE FLUORESCENCE IN EXPERIMENTAL TUMOURS

E. TAPP, R. CARROLL AND K. KOVÁCS
*From the Department of Pathology, The University of Liverpool*

Received for publication February 19, 1965

TETRACYCLINE can be detected in the tissues by its bright yellow fluorescence in ultra-violet light. Using this method it has been shown that, within a few minutes after parenteral administration of the drug, it is distributed to all the tissues of the body except the central nervous system. The highest concentrations are present in the liver, kidneys and bones. At 24 hours the drug has been excreted from all the tissues except the bones where it persists indefinitely in areas of new bone formation (Bottiger, 1955 ; Harris, 1960 ; Carroll and Tapp, 1965).

In 1957 Rall and his associates, while studying the effects of a fluorescent riboflavin antagonist on metastases from carcinoma of the breast in humans, observed at necropsy on patients who had not received the riboflavin antagonist that some other fluorescent material was present in the tumour tissue. Examination of the case records showed that the patients had been given tetracycline some weeks previously. Subsequently these workers reported that a variety of tumours in experimental animals take up and retain tetracycline. Their findings in tumours in man and experimental animals have since been confirmed on many occasions (McLeay, 1958 ; Phillips, Cobb, Richards, Rhodes, Loehrer and Ritchie, 1960 ; Vassar, Saunders and Culling, 1960 ; Málek and Kolc, 1960 ; Milch, Tobie and Robinson, 1961 ; McLeay and Walske, 1962 ; Riley, 1963).

The exact site at which tetracycline is localised in the tumours has been the subject of considerable controversy. Most workers consider that tetracycline is localised only in viable tumour tissue and is absent from necrotic areas (Rall, Loo, Lane and Kelly, 1957 ; Phillips *et al.*, 1960 ; McLeay and Walske, 1962). On the other hand Vassar *et al.* (1960) said that there was no tetracycline in live tumour tissue in human patients, and found it only in macrophages and tissue debris. Very recently Machado, Zaidman, Gerstein, Lichtenberg and Gray (1964) reported that tetracycline was not retained by live cells in transplants of sarcoma-37 in mice but that it was present in the necrotic tumour cells.

In the present studies the uptake and retention of the drug has been investigated in a number of different experimental tumours. Relatively large doses have been used because the subsequent localisation by fluorescent methods is easier.

### MATERIALS AND METHODS

### *The production of tumours*

*Squamous tumours.*—Tumours were produced on both ears of 18 adult rabbits by the biweekly application of 5 drops of a 1% solution of 9,10-dimethyl-1,2-benzanthracene (DMBA) in xylene (Berenblum, 1945).

*Ovarian tumours.*—Tumours were produced in 12 black hooded rats and in 6 albino rats of the Wistar strain by transplanting one ovary into the spleen and excising the remaining ovary (Biskind and Biskind, 1944).

*Mammary tumours.*—Tumours were produced in 24 female albino rats of the Wistar and Sprague-Dawley strains by the administration of 9,10-dimethyl-1,2-benzanthracene (DMBA) in corn oil (Huggins, Briziarelli and Sutton, 1959). The Wistar rats were given the drug by intra-gastric tube in a dose of 10 mg. twice weekly for 3 weeks beginning when the rats were 50 days old. The Sprague-Dawley rats were given a single dose of 50 mg. at 50 days of age.

*Leukaemia.*—Six of the Wistar rats treated with DMBA also developed leukaemia at about the same time that the breast tumours became obvious. A similar finding was reported by Dao (1962) in two out of 100 Sprague-Dawley rats which had received a single dose of 30 mg. of DMBA.

*Tetracycline administration*

Tetracycline hydrochloride was given to the rabbits by intramuscular injection in a dose of 5 mg. per 100 g. body weight. The rats received intramuscular or intraperitoneal injections of 15 mg. per 100 g. body weight or an intravenous injection of 5 mg. per 100 g. body weight. Some animals received a single injection of the drug while others were given four doses at daily intervals. Maximum concentrations of tetracycline are found in the tissues at between one and four hours after the injection. Excretion of the drug occurs rapidly so that at 24 hours very little is found in the body except in the bones. If tetracycline is found in other organs at 48 hours this can be regarded as indicating abnormal retention by the tissues of that organ.

In the present experiments the uptake of tetracycline by the tumours was investigated in animals killed between one and 4 hours after a single injection of tetracycline. The retention of the drug by the tumours was studied in animals killed at times from 2 to 28 days after the last of four injections of tetracycline.

*Histological methods*

Fresh frozen sections cut at 5 $\mu$ were examined microscopically unstained in ultra-violet light. Corresponding frozen sections were stained with haematoxylin and eosin. Paraffin blocks were also prepared from adjacent tissue, and sections cut at 5 $\mu$ were stained with haematoxylin and eosin.

### RESULTS

### *Squamous Tumours*

Small warty growths about 1 cm. in diameter became obvious on the ears of the rabbits between 3 and 4 months after the first application of DMBA. There were usually three or four tumours on each ear. Some of these tumours increased rapidly in size over the next 4 weeks with destruction of the greater part of the ear. Ulceration occurred in many of the tumours and secondary infection of the ulcerated area caused further damage to the ear. A good deal of keratin was present on the surface of some of the tumours.

Although local invasion was marked there was no spread to the local lymph nodes or to the rest of the body.

On histological examination the early lesions were superficial and had a papillomatous growth of squamous epithelium which was heavily keratinised. The large invasive tumours which developed later had the appearances of fairly well differentiated squamous carcinoma. Cell nests containing keratin were present (Fig. 1). In some of the cell nests there were collections of polymorphs and necrotic tumour cells between the layers of keratin, and in other cell nests the whole central mass was a mixture of polymorphs, dead tumour cells and debris. In the non-kertinised parts of the tumour there was also necrosis in the centre of some of the tumour masses (Fig. 2). Invasion of the deeper tissues had occurred, and, although the cartilage appeared to be fairly resistant to invasion, it had been eroded in some places (Fig. 3). Destruction of the cartilage was particularly marked where ulceration and secondary infection was present.

*Tetracycline studies*

There did not appear to be any difference in the amount of tetracycline taken up by the tumours in animals given the 4 day course of tetracycline and in those given only a single dose. The following description applies to both groups.

Only small amounts of tetracycline were taken up by the live tumour cells and even less by the stroma of the tumour. High concentrations were present in thin bands in some keratinised areas (Fig. 4). This was particularly noticeable when the animals had received multiple injections of tetracycline. Much higher concentrations were found in the necrotic cells of the deeper parts of the tumours (Fig. 4) and in the necrotic material in the cell nests (Fig. 5). A good deal of tetracycline was also found in necrotic granulation tissue at the ulcerated surfaces of the tumours (Fig. 6).

Large amounts of tetracycline were retained in necrotic areas and persisted there for at least 4 weeks after the last injection of the drug. A similar retention of tetracycline was also observed in bands of keratin. On the other hand the small

---

EXPLANATION OF PLATES

FIG. 1.—Fairly well differentiated squamous carcinoma with a cell nest containing keratin. H. & E. ×125.

FIG. 2.—Necrosis in the centre of a tumour mass in a non-keratinised part of the tumour. H. & E. ×125.

FIG. 3.—Invasion of the deeper tissues and erosion of the cartilage by the tumour. H. & E. ×125.

FIG. 4.—High concentrations of tetracyline in thin bands in a keratinised part of the tumour and in necrotic cells at the top of the photograph. Unstained frozen section in ultra-violet light. ×80.

FIG. 5.—High concentrations of tetracycline in necrotic material in cell nests. Unstained frozen section in ultra-violet light. ×80.

FIG. 6.—High concentrations of tetracycline at an ulcerated surface. Particularly high concentrations are present in the necrotic granulation tissue (junction of left two thirds and right one third of the photograph). Unstained frozen section in ultra-violet light. ×160.

FIG. 7.—A high power view of the cells in a tumour which was predominently of the granulosa-cell type. H. & E. ×500.

FIG. 8.—Autofluorescence due to lipofuscin pigment in the stroma of an ovarian tumour. Unstained frozen section in ultra-violet light. ×80.

FIG. 9.—Adenocarcinoma of the mammary gland of a rat treated with DMBA. H. & E. ×50.

FIG. 10.—Similar areas of necrosis were present in all the mammary tumours. H. & E. ×50.

FIG. 11.—Tetracycline in a necrotic area of a tumour from an animal killed 3 weeks after the administration of this substance. Unstained frozen section in ultra-violet light. ×50.

FIG. 12.—The immature white blood cells have replaced the normal liver parenchyma in some areas. H. & E. ×500.

1

2

3

4

Tapp, Carroll and Kovács.

5

6

7

8

Tapp, Carroll and Kovács.

9

10

11

12

Tapp, Carroll and Kovacs.

amounts of tetracycline present in the tumour cells and stroma disappeared as the drug was excreted from the rest of the body and there was no evidence that tetracycline was retained in tumour cells which had not undergone necrosis.

### *Ovarian Tumours*

The tumours that developed in the spleen were examined between 6 and 24 months after transplanting the ovary. They were solid tumours, varying between one and four centimeters in diameter, the largest weighing between 40 and 50 g. The colour varied, being mostly greyish-brown but scattered yellowish areas were also present. The tumour had invaded and replaced the greater part of the spleen but there was no invasion of neighbouring organs or spread to other parts of the body.

Histologically the tumours were predominantly of the granulosa-cell type, consisting of large polyhedral-shaped cells arranged in sheets or coarse trabeculae (Fig. 7). The cytoplasm of the cells varied ; usually it was clear and pale but sometimes it was foamy and eosinophilic. The nuclei were round with a stippled chromatin pattern. Some tumours were of mixed type and had a lobular pattern with spindle-shaped cells resembling those found in thecomas between groups of granulosa cells. Granules of a lipfuscin pigment were present amongst the spindle-shaped cells. There was no necrosis in the tumours.

#### *Tetracycline studies*

Tetracycline was taken up in small amounts by the tumour cells of both the granulosa and the coma type but the uptake by the tumour cells was not higher than by normal ovarian tissue. The tetracycline gradually disappeared from these cells during the 48 hours after the injection of the drug. There was no retention of tetracycline by the tumour.

A substance which gave a dull orange-yellow autofluorescence, quite distinct from the bright yellow fluorescence of tetracycline, was present in the stroma of the tumour (Fig. 8). This was found to be the lipofuscin pigment noted in the stained sections.

### *Mammary Tumours*

These tumours developed between 6 and 16 weeks from the beginning of the course of DMBA and were studied about 2 to 3 weeks later, by which time they had grown to between 2 and 3 cm. in diameter. In some animals tumours developed in more than one mammary gland. The consistency of the tumours was in general firm although there were small areas of softening. The cut surface was greyish-white in colour.

Histologically the tumours had the structure of adenocarcinomas but there was considerable variation in the degree of differentiation even in the same tumour (Fig. 9). In the more highly differentiated parts there were tubules lined by a single layer of columnar epithelium in which there were only occasional mitoses. These tubules were separated by a stroma containing spindle-shaped cells and chronic inflammatory cells. In some parts of these areas a " comedo " pattern could be seen.

In less differentiated areas tubule formation was still seen but the acini were lined by several layers of cells which varied in type from tall columnar to flattened epithelium. Mitoses were relatively common in this epithelium. There was much less stroma between the tubules.

Other areas were more anaplastic with solid sheets of cells in which there were many mitotic figures.

Areas of necrosis of variable extent were present in all the tumours and were particularly marked in the less differentiated ones (Fig. 10).

*Tetracycline studies*

There was a diffuse low uptake of tetracycline by the tumour cells, comparable with the amount found in normal epithelial cells of the mammary gland. There did not appear to be any difference in the amount of tetracycline taken up by tumour cells of varying degrees of differentiation. Very little tetracycline was taken up by the stroma. In contrast large amounts of the drug were found in necrotic areas of the tumours. Here the tetracycline was seen to be in the cytoplasm of necrotic tumour cells but the appearances varied depending on the extent of the histological changes. In cells which had lost their nuclei but whose cytoplasm had not become very dense there were large clumps of tetracycline. On the other hand where the tumour cells were shrunken and had begun to disintegrate they were often admixed with the remains of polymorphs and macrophages, and here the tetracycline appeared to be scattered in and amongst the cells in a fine powder. The powdery appearance was also seen in the inspissated eosinophilic material which filled the acini in the " comedo " type of tumour.

Tetracycline was retained in these necrotic areas for at least four weeks (Fig. 11). There was no retention of the drug by live tumour cells.

### *Leukaemia*

Some of the rats treated with DMBA by stomach tube became very anaemic. Peripheral blood films were found to contain large numbers of immature white blood cells of the type found in stem cell leukaemia.

At post-mortem examination the liver and spleen were grossly enlarged and there was obvious enlargement of lymph nodes. In occasional animals the kidneys contained discrete nodules of tumour tissue 1–2 mm. in diameter.

Histologically there was a diffuse infiltration of the liver by immature white blood cells which had replaced the normal liver parenchyma in some areas (Fig. 12). The normal structure of the spleen and of the lymph glands was destroyed by an extensive infiltration with leukaemic cells. In the kidney the discrete nodules were found to be composed of masses of leukaemic cells which had completely replaced the normal tissues. Microscopic foci of leukaemic cells were also found in the cortex of the adrenal glands.

*Tetracycline studies*

There was no uptake of tetracycline by the leukaemic cells either in the peripheral blood or in the liver, spleen, kidney or adrenal glands. The areas containing leukaemic deposits in the liver and kidney appeared dark against the bright fluorescence caused by the presence of high concentrations of tetracycline in the normal tissues of these organs.

### *Experimentally Produced Necrosis in Tumours*

It appears from the above observations that tetracycline is taken up by necrotic tumour cells and necrotic debris in the tumours. For this reason further studies were carried out on the mammary tumours after attempts had been made to increase the amount of necrosis in the tumours.

*Methods.*—Small tumours about 0·7 cm. in diameter were selected for these experiments as these tumours have been found to have undergone little if any necrosis and consequently only take up small amounts of tetracycline. Two types of experiments were carried out.

(a) The tumour was dissected at operation and a temporary ligature tied around its main blood vessels. In some animals the ligature was removed after one hour while in others it was left in place for 2 hours. Tetracycline was given at 24 hours and the animals were killed one hour later.

(b) The tumour was removed at operation and a small piece of it was transplanted into the spleen of the same or another animal. Tetracycline was given at 10 days and the animals were killed one hour later.

*Results.*—In stained sections there were areas of necrosis of varying sizes in the ligated tumours. The greater part of the transplanted pieces of tumour were also necrotic although some of the cells at the edge were still alive. Some of the large necrotic areas had the typical appearances of large infarcts with a central dead area and the usual margin zones (Sheehan and Davis, 1958). Smaller necrotic areas were composed only of margin zones.

The tetracycline had been taken up by the necrotic tumour cells in the margin zones of the large infarcts but not in the central dead area. In the smaller lesions it was seen in the necrotic tumour cells throughout the area of necrosis. The exact appearances varied as in the spontaneous necroses described previously, but usually it was present as large clumps in the necrotic tumour cells.

*Comment.*—It is clear from these experiments that tumour cells which under normal circumstances do not take up large amounts of tetracycline do so when they undergo necrosis. The failure of the necrotic tumour cells in the central dead areas of large infarcts to take up tetracycline is explicable because this area does not have a circulation and is not subject to diffusion (Carroll and Tapp, 1965).

### DISCUSSION

The most constant feature in these studies was the high uptake of tetracycline by necrotic tumour cells and other debris. The drug was retained in these areas for at least 4 weeks. The live tumour cells took up only the same amounts as normal tissues and did not retain it for more than 24 hours. This applied to all the tumour cells irrespective of their degree of differentiation.

Rall *et al.* (1957), McLeay (1958) and Phillips *et al.* (1960) reported that tetracycline appeared to be taken up by live tumour tissue and not by the necrotic or haemorrhagic parts, but their observations were based only on macroscopic examination. More recently McLeay and Walske (1962) have reported that preliminary microscopic studies indicate that tetracycline is localised in the cytoplasm of live tumour cells, but they do not give any details of the methods they used. Their results are not in agreement with the present findings.

The possible diagnostic and therapeutic uses of tetracycline uptake by tumours have been discussed recently by Berk (1963). As tetracycline was considered to be localised in the live tumour cell it was suggested that tetracycline might be of value in distinguishing between normal and neoplastic areas especially at easily accessible sites in the body. Lipnik (1963) applied a solution of dimethylchlortetracycline to various skin lesions, and found that they all took up the substance. When he then treated the area with trichloracetic acid the substance was removed from benign lesions but not from malignant lesions which thus remained fluorescent. He did however report false positive results in cases of benign tumours which were inflamed and in crusted and healing lesions of impetigo. Cholewa, Konturek and Gróski (1963) administered tetracycline systemically to patients with tumours of the cervix and lip, and were able to detect the drug in the tumours by macroscopic inspection in ultra-violet light. In view of the present findings it is unlikely that these local or general techniques show anything more than the presence of necrosis in tumours.

Tetracycline has been found to persist longer in the gastric contents of patients with carcinoma of the stomach than in patients with benign lesions of the stomach and in normal control persons. Klinger and Katz (1961) Berk and Kantor (1962) Sandlow, Allen and Necheles (1963) and Sherman, Chryssanthou, Sukoff, Mininberg, Beckman and Weingarten (1963) said that this test was of diagnostic value for malignant lesions of the stomach but Aberle (1963), Rugtveit and Hope (1964) and Cummins, Gompertz and Kier (1964) have reported a large number of false positive tests in patients with benign lesions of the stomach. It is clear from the present study that benign lesions such as chronic gastric ulcers with areas of necrosis might well retain tetracycline and thus give false positive results.

The urinary excretion of tetracycline following a single dose has been reported to be prolonged in patients with carcinoma (Cholewa *et al.*, 1963 ; Morador, 1963) and this has been suggested as a diagnostic test. When the non-specific nature of tetracycline fixation to necrotic tissue is considered, such a test may be merely a measure of the amount of necrotic tissue in the body.

Any drug which localises in tumour tissue has possible therapeutic applications, and certain workers have therefore combined tetracycline with radioactive substances for the treatment of experimental tumours (Dunn, Eskelson, McLeay, Ogborn and Walske, 1960 ; Phillips *et al.*, 1960). McLeay, Walske and Ogborn (1960) found good uptake of an $^{131}I$ labelled tetracycline compound in spontaneous mammary tumours in mice, and considered that the radioactive compound resulted in necrosis of these tumours. The present work suggests that the uptake will only occur in tumours which have areas of necrosis before the treatment is instituted.

The uptake of tetracycline by necrotic cells of tumours is in keeping with the findings in pathological conditions of the liver and kidney. In these organs, cells which had become necrotic due to experimental ischaemia or toxic chemicals were found to take up tetracycline in large amounts and to retain it for considerably longer than the normal cells (Tapp, Carroll and Kovács, 1964 ; Carroll and Tapp, 1965). In the necrotic areas of the liver after thioacetamide or carbon tetrachloride poisoning there are large amounts of calcium and the tetracycline accumulation in such necrotic areas is closely related to its calcium content (Tapp and Carroll, 1965). It has been known for some years that necrotic areas in tumours also contain much higher concentrations of calcium than live tumour tissue (Shear, 1933,; Suntzeff and Carruthers, 1944) and it is possible that this in a similar way may account for the accumulation of tetracycline in such areas.

It was observed in the squamous tumours that keratin became labelled with tetracycline in much the same way as bone. This finding is of interest in view of the report that tetracycline is present in the finger nails of chronic bronchitics treated with the drug for long periods (Douglas, 1963).

SUMMARY

The uptake and retention of tetracycline was studied in a number of different types of experimental tumours including stem cell leukaemia.

The tetracycline was taken up in large amounts by necrotic tumour tissue and was retained there for a long period. The uptake and retention by live tumour cells was comparable to that in normal tissues.

When necrosis was produced in growing tumours by temporary ligature of the blood supply or transplantation to the spleen, there was a greater uptake of tetracycline localised to the areas of necrosis.

We wish to thank Professor H. L. Sheehan for his helpful advice and criticism during the course of the experimental work and in the preparation of the paper.

Dr. K. Kovács wishes to thank the Crosby Cancer Research Fund for a Fellowship held during the course of this work.

### REFERENCES

Aberle, S.—(1963) *Gastroenterology*, **44**, 933.
Berenblum, I.—(1945) *Cancer Res.*, **5**, 265.
Berk, J. E.—(1963) *Curr. ther. Res.*, **5**, 331.
*Idem* and Kantor, S. M.—(1962) *J. Amer. med. Ass.*, **179**, 997.
Biskind, M. S. and Biskind, G. S.—(1944) *Proc. Soc. exp. Biol. Med.*, **55**, 176.
Bottiger, L. E.—(1955) *Antibiotics Chemother.*, **5**, 332.
Carroll, R. and Tapp, E.—(1965) *J. Path. Bact.*, **89**, 35.
Cholewa, L., Konturek, S. and Gróski, L.—(1963) *Bull. Pol. med. Hist. Sci.*, **6**, 51.
Cummins, A. J., Gompertz, M. L. and Kier, J. H.—(1964) *Ann. intern. Med.*, **61**, 56.
Dao, T. L.—(1962) *Cancer Res.*, **22**, 973.
Douglas, A. C.—(1963) *Brit. J. Dis. Chest.*, **57**, 44.
Dunn, A. L., Eskelson, C. D., McLeay, J. F., Ogborn, R. E. and Walske, B. R.—(1960) *Proc. Soc. exp. Biol. Med.*,**104**, 12.
Harris, W. H.—(1960) *Nature, Lond.*, **188**, 1038.
Huggins, S. C., Briziarelli, G. and Sutton, H. Jr.—(1959) *J. exp. Med.*, **109**, 25.
Klinger, J. and Katz, R.—(1961) *Gastroenterology*, **41**, 29.
Lipnik, M. J.—(1963) *Archs Derm.*, **87**, 575.
Machado, L., Zaidman, I., Gerstein, J. F., Lichtenberg, F. and Gray, S. J.—(1964) *Cancer Res.*, **24**, 1845.
McLeay, J. F.—(1958) *Am. J. Surg.*, **96**, 415.
*Idem* and Walske, B. R.—(1962) *Ann. Surg.*, **156**, 313.
*Iidem* and Ogborn, R. E.—(1960) *Surg. Forum*, **11**, 79.
Málek, P. and Kolc, J.—(1960) *Antibiotics Chemother.*, **10**, 488.
Milch, R. A., Tobie, J. E. and Robinson, R. A.—(1961) *J. Histochem. Cytochem.*, **9**, 261.
Morador, J. L.—(1963) *Semana med.*, B. Aires, **122**, 1028.
Phillips, J. W., Cobb, E. G., Richards, V., Rhodes, W. D., Loehrer, D. C. and Ritchie, J. L.—(1960) *Am. J. Surg.*, **100**, 384.
Rall, D. P., Loo, Ti. Li., Lane, M. and Kelly, M. G.—(1957) *J. nat. Cancer Inst.*, **19**, 79.

RILEY, L. H. JR.—(1963) *Johns Hopkins Hosp. Bull.*, **113**, 291.
RUGTVEIT, A. AND HOPE, L.—(1964) *Gastroenterology*, **47**, 32.
SANDLOW, L. J., ALLEN, H. A. AND NECHELES, H.—(1963) *Ann. intern. Med.*, **58**, 409.
SHEAR, M. J.—(1933) *Am. J. Cancer*, **18**, 924.
SHEEHAN, H. L. AND DAVIS, J. C.—(1958) *J. Path. Bact.*, **76**, 569.
SHERMAN, H. H., CHRYSSANTHOU, C., SUKOFF, M. H., MININBERG, D., BECKMAN, E. M. AND WEINGARTEN, M.—(1963) *Gastroenterology*, **45**, 84.
SUNTZEFF, V. AND CARRUTHERS, C.—(1944) *J. biol. Chem.*, **153**, 521.
TAPP, E., CARROLL, R. AND KOVÁCS, K.—(1964) *Experientia*, **20**, 393.
TAPP, E. AND CARROLL, R.—(1965) *J. Path. Bact.*, **89**, 715.
VASSAR, P. S., SAUNDERS, A. M. AND CULLING, C. F. A.—(1960) *Archs Path.*, **69**, 613.


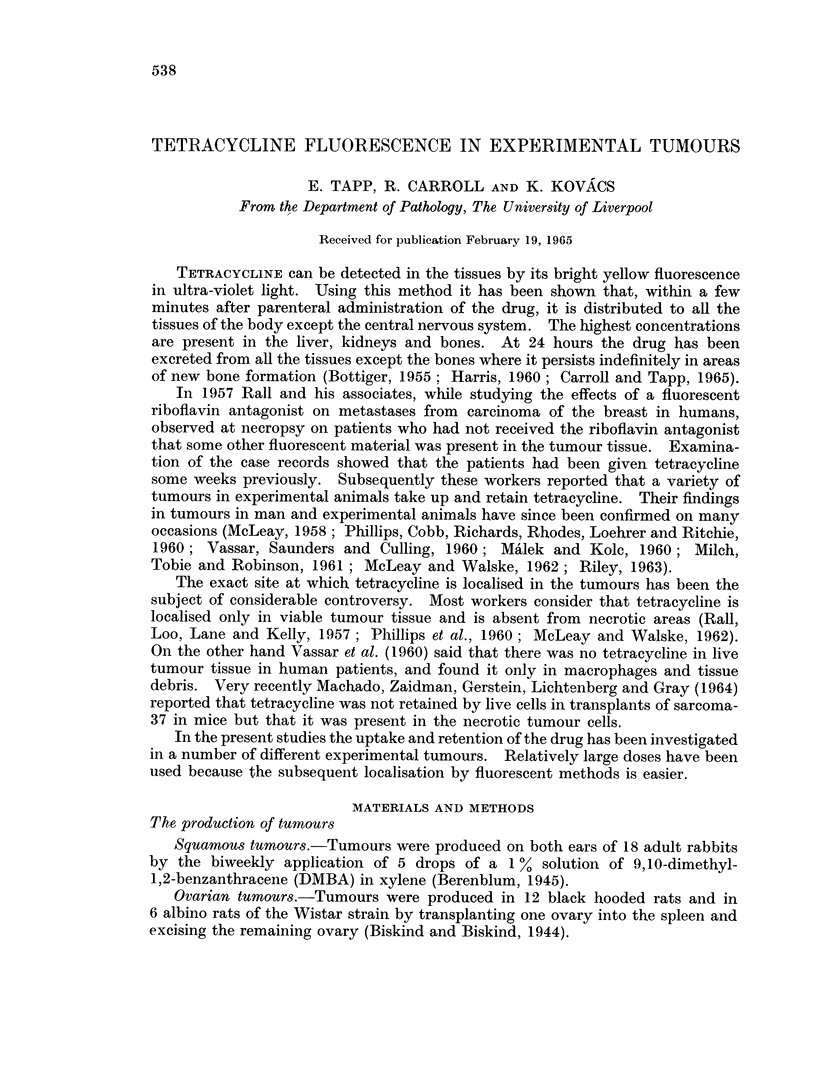

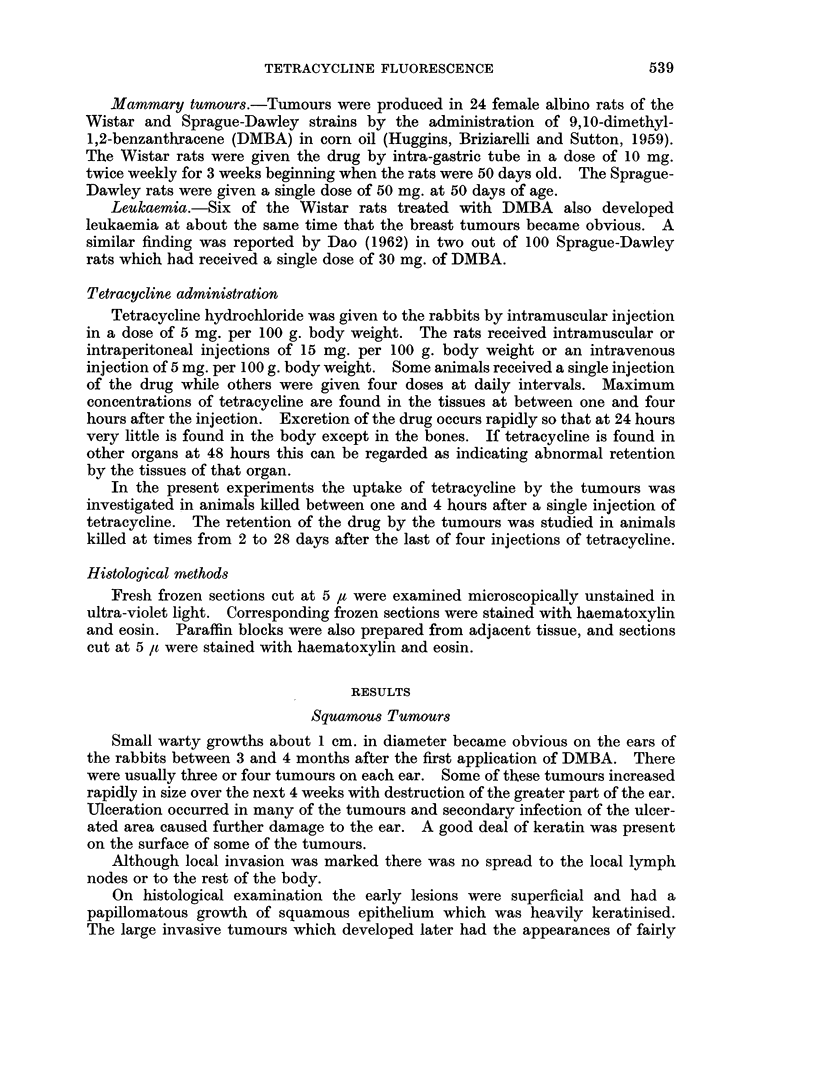

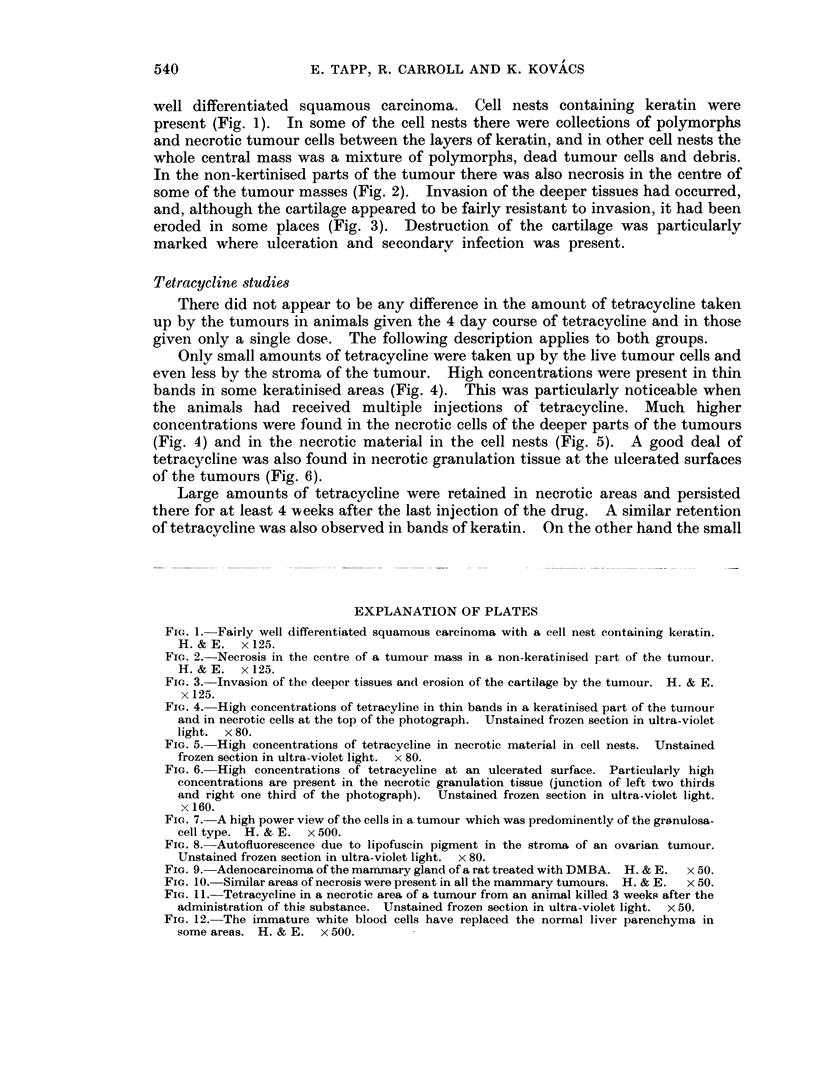

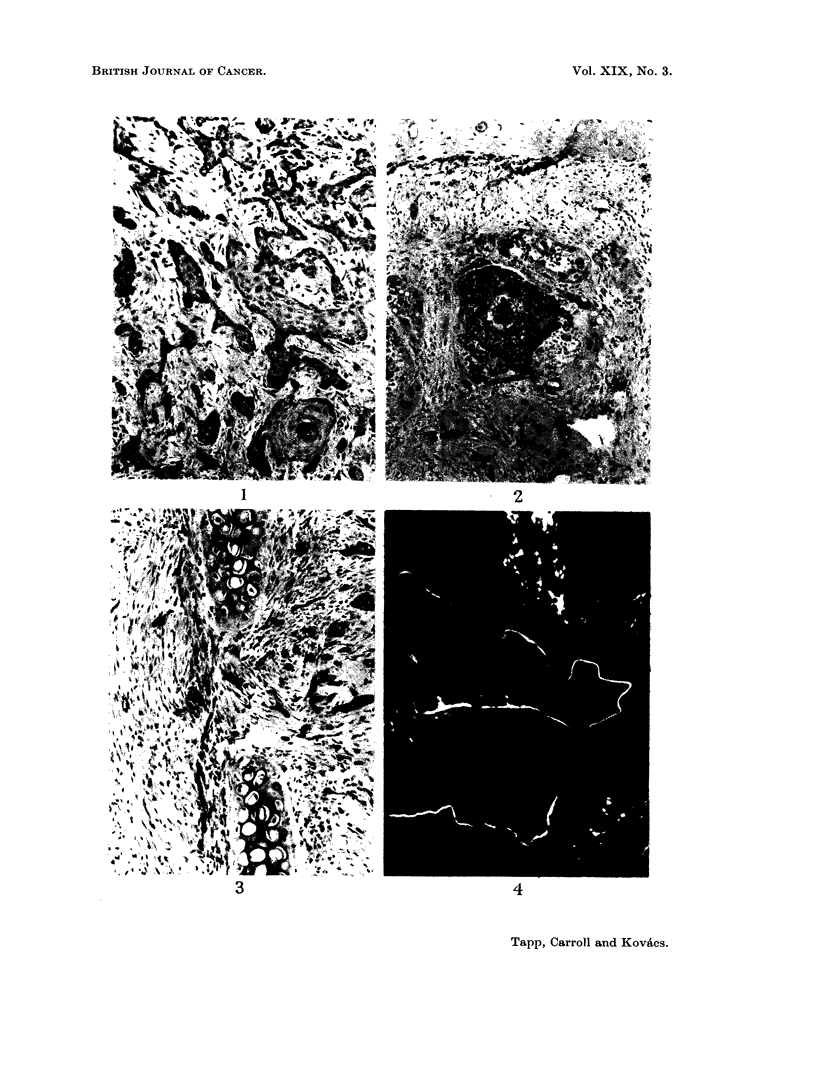

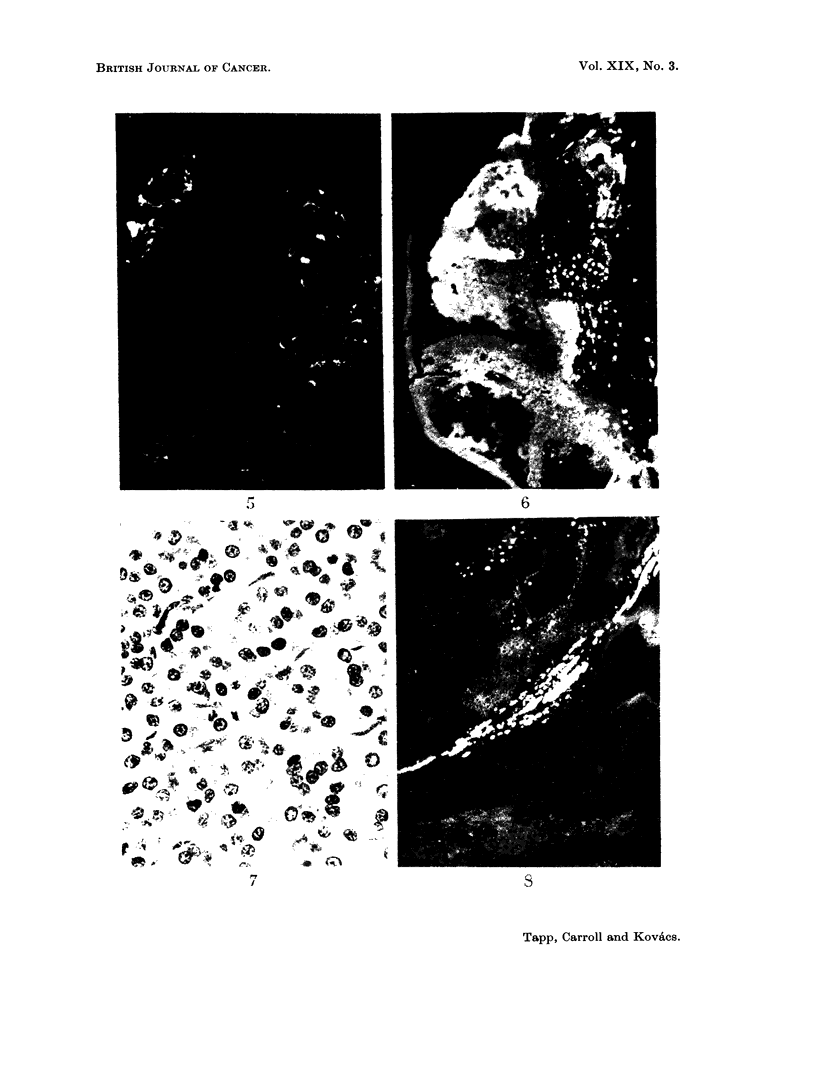

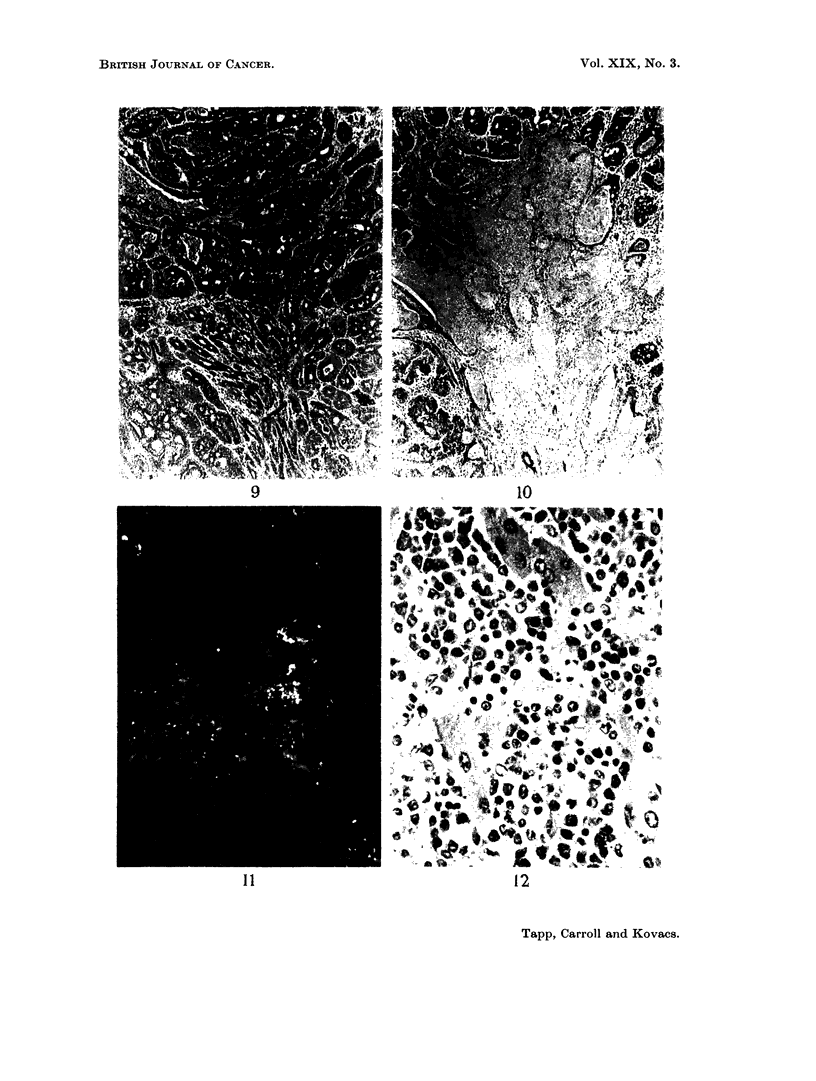

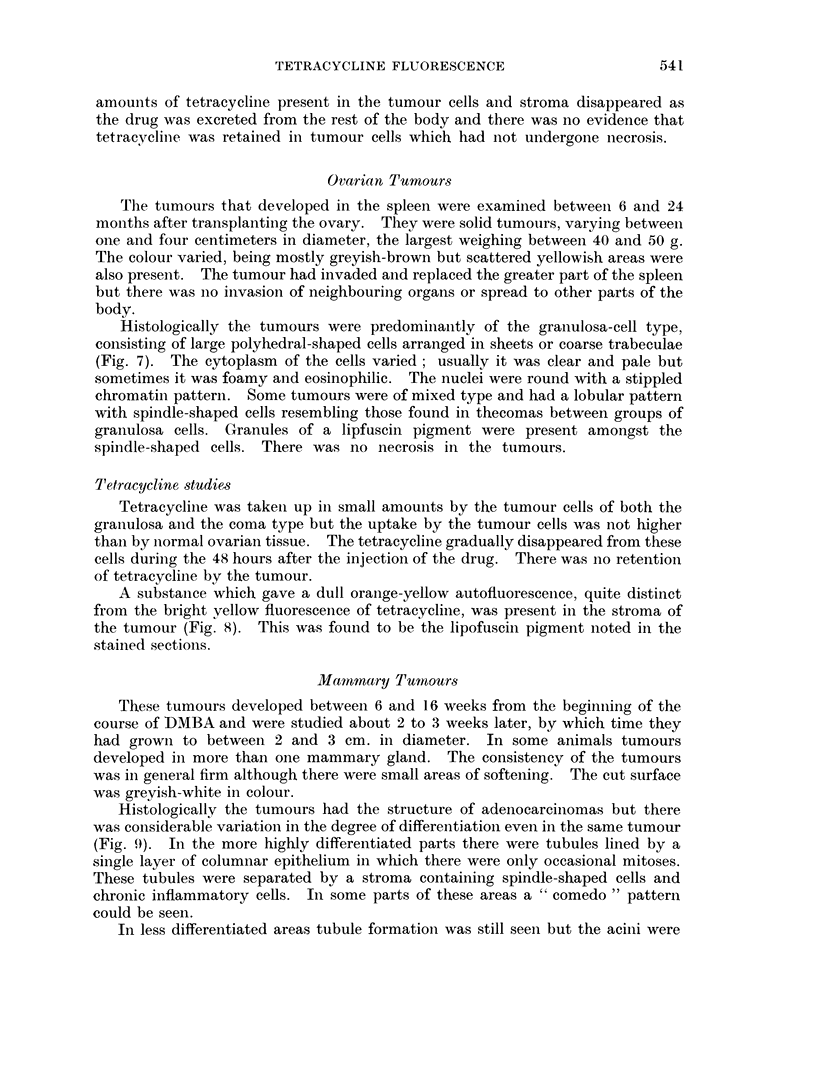

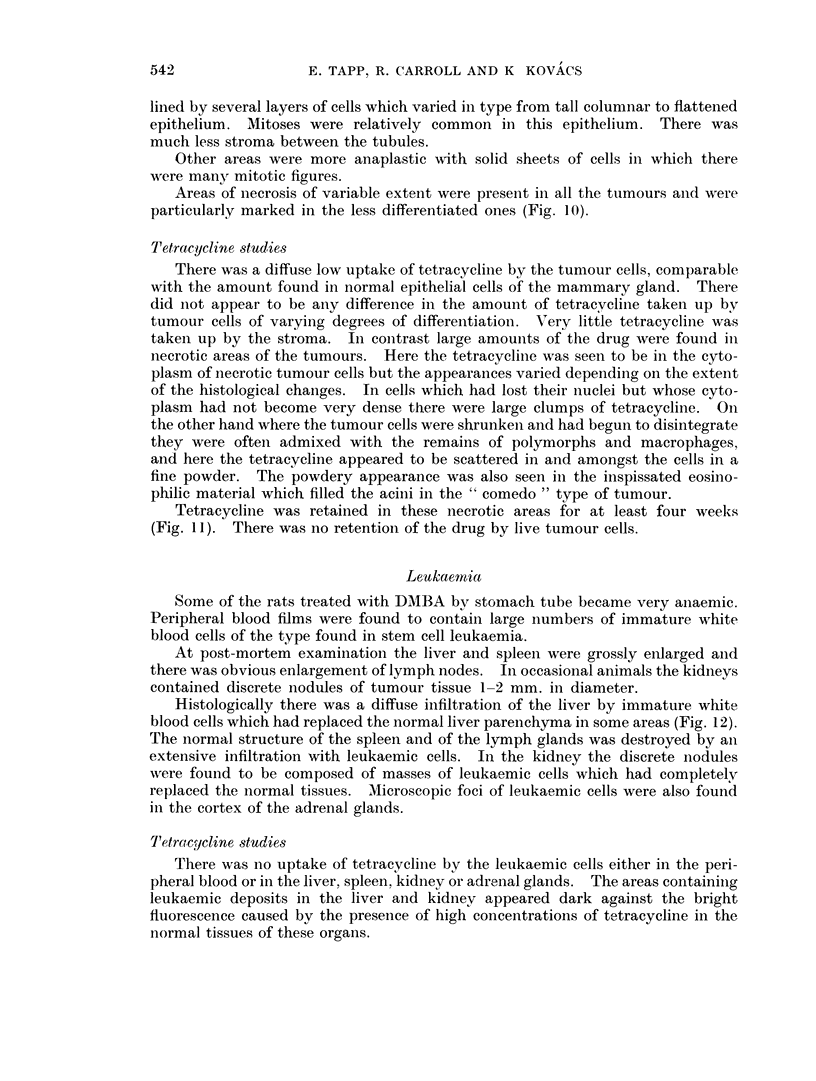

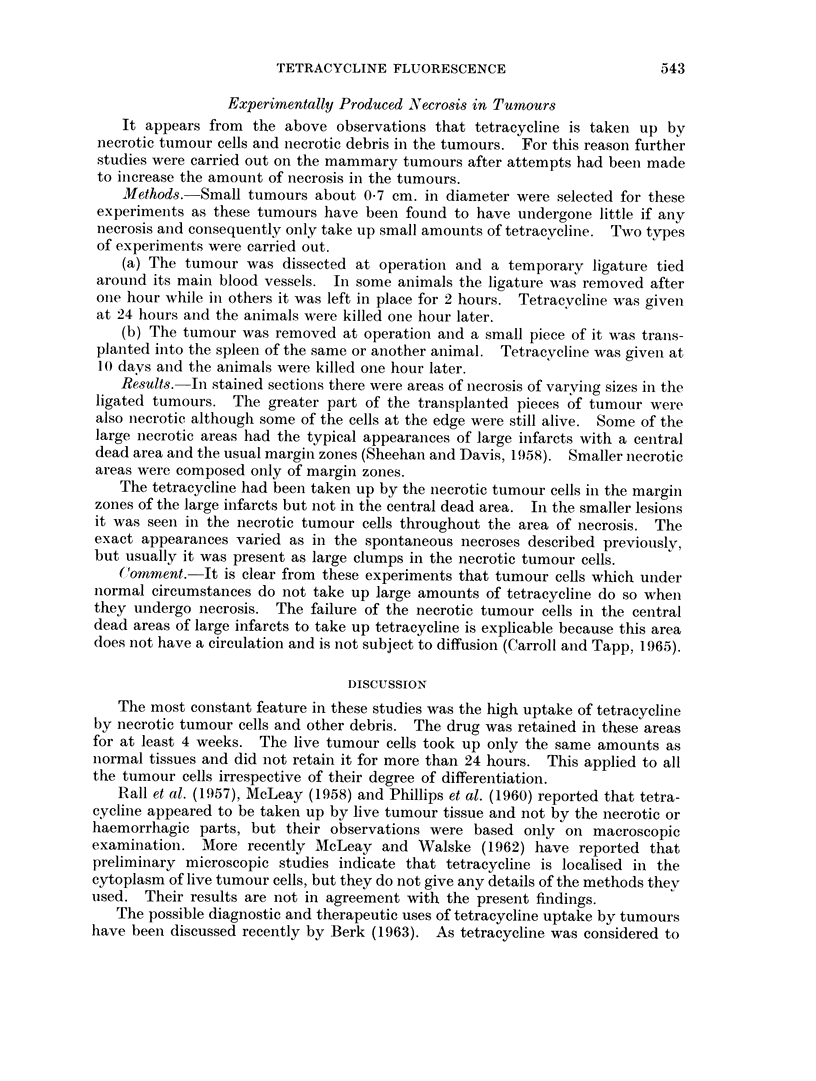

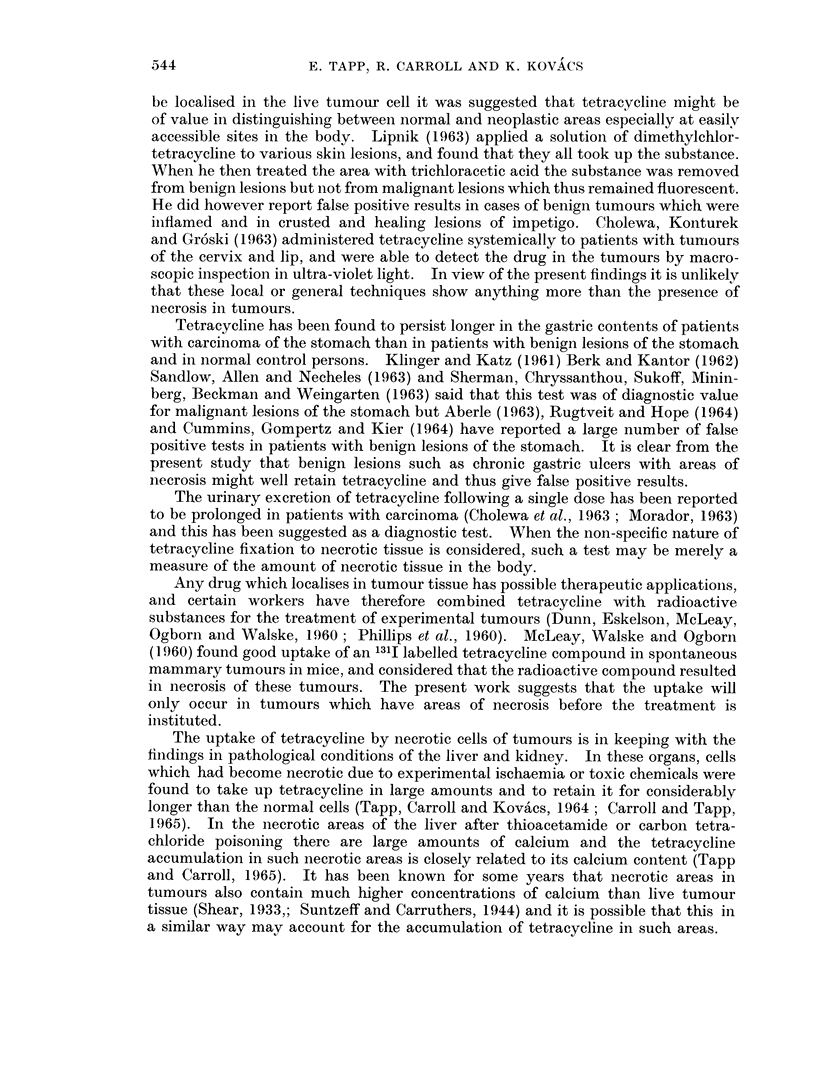

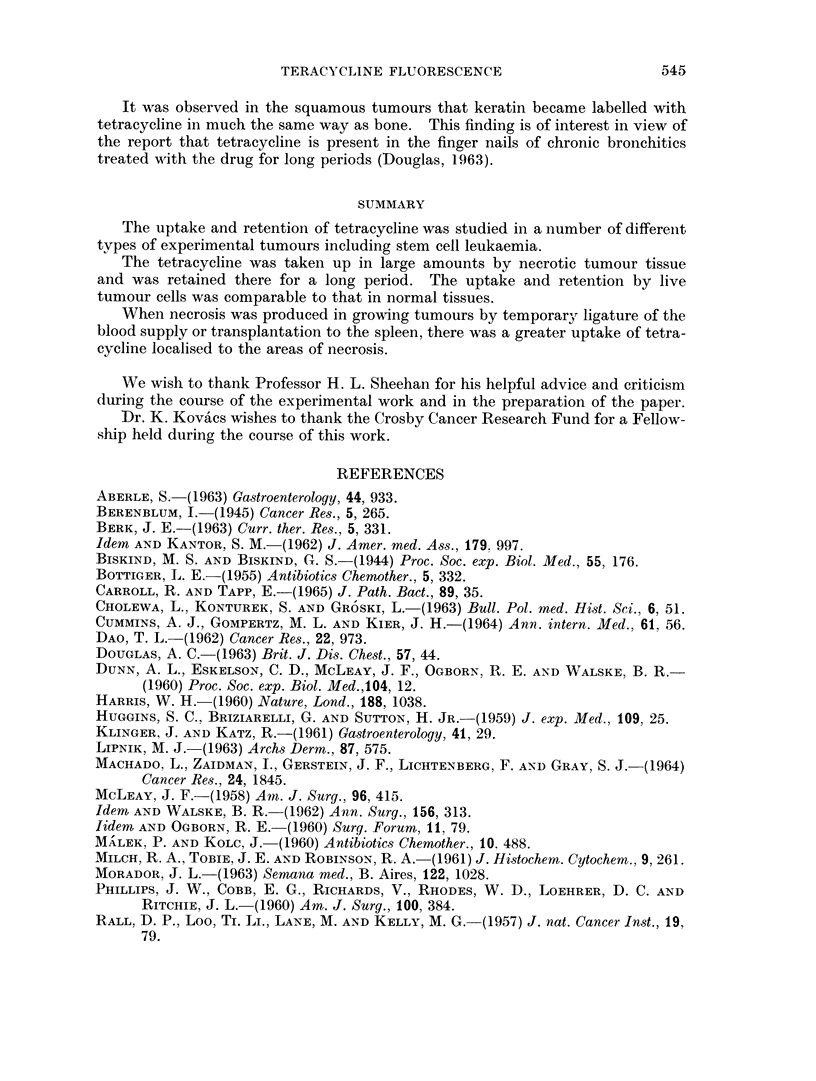

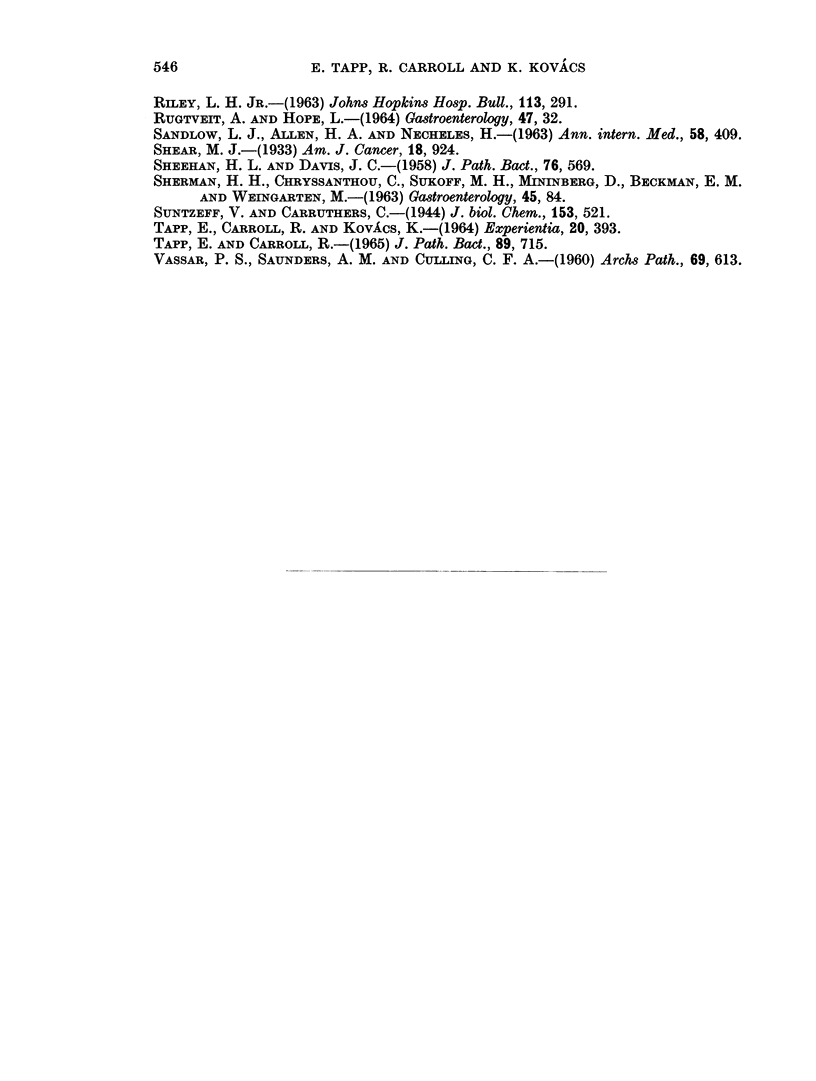


## References

[OCR_00504] ABERLE S. (1963). The tetracycline fluorescence test in differential diagnosis of gastric disease.. Gastroenterology.

[OCR_00508] BERK J. E., KANTOR S. M. (1962). Demethylchlortetracycline-induced fluorescence of gastric sediment. Use to differentiate benign and malignant gastric lesions.. JAMA.

[OCR_00506] BERK J. E. (1963). Tetracycline fluorescence: a serendipitous gain.. Curr Ther Res Clin Exp.

[OCR_00517] DAO T. L. (1962). The role of ovarian hormones in initiating the induction of mammary cancer in rats by polynuclear hydrocarbons.. Cancer Res.

[OCR_00531] MACHADO L., ZAIDMAN I., GERSTEIN J. F., LICHTENBERG F., GRAY S. J. (1964). FACTORS AFFECTING THE SITE AND DEGREE OF LOCALIZATION OF TETRACYCLINE IN SARCOMA 37 TUMORS.. Cancer Res.

[OCR_00540] MALEK P., KOLC J. (1960). Penetration of chlortetracycline into tissue affected by pathological changes.. Antibiot Chemother (Northfield).

[OCR_00542] MILCH R. A., TOBIE J. E., ROBINSON R. A. (1961). A microscopic study of tetracycline localization in skeletal neoplasms.. J Histochem Cytochem.

[OCR_00535] McLeay J. F., Walske B. R. (1962). Relationship of Tetracycline to Carcinoma.. Ann Surg.

[OCR_00545] PHILLIPS J. W., COBB E. G., RICHARDS V., RHODES W. D., LOEHRER D. C., RITCHIE J. L. (1960). The deposition and retention of tetracycline in cancer.. Am J Surg.

[OCR_00556] RILEY L. H. (1963). TETRACYCLINE INDUCED FLUORESCENCE IN A TRANSPLANTED HUMAN TUMOR.. Bull Johns Hopkins Hosp.

[OCR_00558] SANDLOW L. J., ALLEN H. A., NECHELES H. (1963). The use of tetracycline fluorescence in the detection of gastric malignancy.. Ann Intern Med.

[OCR_00561] SHEEHAN H. L., DAVIS J. C. (1958). Complete permanent renal ischaemia.. J Pathol Bacteriol.

[OCR_00570] TAPP E., CARROLL R. (1965). TETRACYCLINE ACCUMULATION IN TOXIC LIVER DAMAGE.. J Pathol Bacteriol.

[OCR_00569] Tapp E., Carroll R., Kovács K. (1964). The use of tetracycline fluorescence to identify necrosis of renal tubules and to assess renal blood flow.. Experientia.

[OCR_00572] VASSAR P. S., SAUNDERS A. M., CULLING C. F. (1960). Tetracycline fluorescence in malignant tumors and benign ulcers.. Arch Pathol.

